# The change and correlates of healthy ageing among Chinese older adults: findings from the China health and retirement longitudinal study

**DOI:** 10.1186/s12877-021-02026-y

**Published:** 2021-01-27

**Authors:** Peng Nie, Yan Li, Nan Zhang, Xiaomin Sun, Bao Xin, Youfa Wang

**Affiliations:** 1 School of Economics and Finance, Xi’an Jiaotong University, Xi’an, China; 2 Institute for Health Care & Public Management, University of Hohenheim, 70599 Stuttgart, Germany; 3 Global Health Institute, School of Public Health, Xi’an Jiaotong University Health Science Center, Xi’an, Shaanxi China; 4 Manchester Institute for Collaborative Research on Ageing (MICRA), Social Statistics, School of Social Sciences, The University of Manchester, Manchester, UK; 5 Department of Food Hygiene and Nutrition, School of Public Health, Shaanxi University of Chinese Medicine, Xianyang, Shaanxi China

**Keywords:** Healthy ageing, Older adults, Spatial characteristics, China

## Abstract

**Background:**

This study tentatively constructs a composite measure of Chinese Healthy Ageing Index (CHAI) among older adults aged 60+ and investigates change of CHAI during 2011–2015 and its association with sociodemographic characteristics.

**Methods:**

Data collected from 8182 old adults aged 60+ in the 2011 and 2015 China Health and Retirement Longitudinal Study (CHARLS, a nationally representative sample) were used. Six medical measures of blood pressure, peak expiratory flow, cognitive status score, fasting glucose, kidney function and C-reactive function were used to construct CHAI (range 0–12, 0–2 = healthiest, 7–12 = unhealthiest). Ordinary least squares, logistic and random effects models examined social and spatial determinants of CHAI score and the prevalence of the ideal CHAI. Unconditional quantile regression tested heterogeneous impacts of sociodemographic determinants of CHAI score.

**Results:**

Mean CHAI score declined from 5.7 to 5.2, and the proportion of the ideal CHAI (CHAI score = 0–2) increased from 5.6 to 9.4% during 2011–2015, indicating an improvement in healthy ageing over time. During 2011–2015, the highest rates of the ideal CHAI were in Southeast and East of China. Older adults, male, living in the Center and West, smoking, obesity/overweight and having chronic diseases were positively associated with total CHAI score and negatively with a higher prevalence of the ideal CHAI. Being married, having high education and regular social activities were associated with a higher rate of the ideal CHAI. The positive predictors for total CHAI were stronger in those with worse CHAI status.

**Conclusions:**

In China healthy ageing has improved during 2011–2015, but substantial geographical and sociodemographic heterogeneities exist in the improvements, suggesting health equality remains a challenge in China. Future policies and interventions should especially focus on men, those in Central and West China, and combat health problems like obesity, chronic diseases and unhealthy behaviors.

**Supplementary Information:**

The online version contains supplementary material available at 10.1186/s12877-021-02026-y.

## Background

Population ageing is a global challenge and affects both developed and developing countries, whilst its pace has been fastest in Asia [1] especially in Eastern and South-Eastern Asia [2]. Such continued rapid population ageing presents challenges for Sustainable Development Goals (SDGs), in particular, ensuring healthy lives and well-being at all ages (SDG 3). China already has the world’s largest ageing population and is one of the fastest ageing societies worldwide. In 2019, 254 million people were aged 60 and over (18.1% of the total population) in China [3] and this number is projected to reach 491.5 million (36.5% of the total population) by 2050 [2]. Healthy ageing, defined as “process of developing and maintaining the functional ability that enables well-being in older age” [4], has become an important theme for the world, especially in less developed countries like China where the population is ageing unprecedentedly. However, the evidence based on an improved health span (the length of time an individual is able to maintain good health) [5] is less encouraging, highlighting the urgency for research on healthy ageing.

To assess well the older adults’ health status and related factors is important and useful to provide them with related care and assist others (e.g., health serve professionals, care providers, and family members) to take related actions. To develop some related assessment tool would be useful. The Healthy Ageing Index (HAI) is one of such index scores, and can help serve this part of this goal. HAI was a modified Physiological Index of Comorbidity [6], was developed to cover a broad range of health indicators over multiple organ systems and capture subclinical decrements in function [7]. HAI had been developed in the Western countries, and was a valid predictor for mortality, disability, and cardiovascular diseases among older adults [6–13]. In particular, Dieteren et al. [13] studied development in individual HAI scores over the life course among people aged between 30 and 70 years in the Netherlands. A recent study constructed the Chinese Healthy Ageing Index (CHAI, range 0–12, lower score was better), and shown that mean CHAI score was 5.6 in 2011, and that the younger, more educated and married people were more likely to have a higher prevalence of “ideal CHAI” (0–2) [14]. Furthermore, CHAI alone predicted mortality substantially better than chronic conditions alone, suggesting that CHAI is useful for predicting death and disability in Chinese older adults and also be valuable in pinpointing those at very low risk of adverse outcomes and the potential to survive to very old age [14]. However, limited related research has been conducted in China, and few studies monitored the change of CHAI and what factor might affect the changes of CHAI over time.

Using data from a large national sample included in the 2011 and 2015 China Health and Retirement Longitudinal Study (CHARLS), the present study aimed to: (1) track change of the CHAI among Chinese older adults during 2011–2015; (2) examine CHAI differences by sociodemographic characteristics; (3) identify geographic heterogeneities in the rates of the ideal CHAI over time; and (4) explore the impacts of demographic and socioeconomic characteristics on CHAI. Our findings will help identify older adults with exceptional health and uncover healthy ageing-related predictors, which may be useful for promoting healthy and productive longevity for Chinese older adults.

## Methods

### Study design and study population

The data were draw from the CHARLS, a nationally representative longitudinal survey of community-living adults aged 45 and over in China. The CHARLS sample was obtained via multistage stratified probability proportional to size sampling design [15]. At national baseline survey (between June 2011 and March 2012), 17,708 respondents in 10,257 households were interviewed. Two follow-up interviews were conducted in 2013 and 2015 [16]. CHARLS collected and assayed venous blood samples in 2011 and 2015 waves. Analysis of blood samples encompassed 2 stages: (1) a complete blood count analysis was performed at local county health center after collection; and (2) the samples were all then transferred to the study headquarters where they were assayed [17].

The ethical review committee at Peking University (IRB 00001052–11,014) approved CHARLS for the biomarker sample collection. The analytic sample used for this present study was restricted to adults aged 60 and over for whom detailed demographic, socioeconomic, and biomarker information was available for two waves. The final sample size was 8182; 3695 and 4487 in the CHARLS 2011 and 2015, respectively.

### Construction of the CHAI

Following Wu et al. [14], we also used the same six key physiologic domains, including systolic blood pressure (SBP), pulmonary function, fasting glucose and cognitive function, kidney function and high-sensitivity C-reactive protein (hsCRP), to generate the CHAI. The choice of each component in the original physiologic index was based on research that identified each as a key predictor of mortality and as a primary measure of a common age-associated chronic disease [18, 19]. Specifically, six physiologic domains included:

1. *SBP*: SBP was measured by three times with 45 s interval and the mean was adopted in our analysis. SBP was grouped into three categories: 0 = ≤120 mmHg, 1 = 120–140 mmHg and 2 = > 140 mmHg. Respondents who were diagnosed by hypertension or were taking anti-hypertensive medications were treated as the unhealthiest group (score = 2) [14].
2. *Pulmonary function*: Pulmonary function was measured by expiratory peak flow (L/min) through a peak flow in the standing position. We used three measurements of expiratory peak flow and calculated the mean value. We then used sex-specific tertiles to categorize it into three groups (for males: 0 = ≥320 L/min, 1 = 193–320 L/min and 2 = ≤ 193 L/min; for females: 0 = ≥225 L/min, 1 = 153–225 L/min and 2 = ≤ 153 L/min) [14]. Respondents who were diagnosed by pulmonary disease were grouped as 2, denoting the unhealthiest.
3. *Fasting glucose*: Fasting glucose was grouped into three categories: 0 = ≤100 mg/dL, 1 = 100–125 mg/dL and 2 = ≥125 mg/dL [14]. Respondents who were diagnosed by diabetes or were taking any anti-diabetic medications were categorized as the unhealthiest group.
4. *Cognitive function*: Cognitive function was assessed by the Telephone Interview for Cognitive Status (TICS). CHARLS encompassed two cognition measures – episodic memory and mental intactness. Episodic memory was evaluated by asking respondents to immediately repeat in any order 10 Chinese nouns just read to them (immediate word recall), and a delayed recall the same list of words 4 min later (delayed recall). Mental intactness was assessed by asking respondents to name the date, the day of the week, re-draw a formerly shown photo, and serial 7 subtraction from 100 (up to five times) [20]. The validity of the TICS was confirmed in different populations [20–22]. Following Wu et al. [14], we grouped the TICS scores into three categorizations (for males: 0 = ≥19, 1 = 14–19 and 2 = ≤ 14; for females: 0 = ≥17, 1 = 10–17 and 2 = ≤ 10).
5. *Kidney function*: Kidney function was assessed by an estimated glomerular filtration rate (eGFR) [23, 24]. We then adopted clinically relevant cutoffs to group eGFR into three categories: 0 = ≥ 90 mL/min per 1.73 m^2^, 1 = 60–90 mL/min per 1.73 m^2^ and 2 = < 60 mL/min per 1.73 m^2^ [14].
6. *hsCRP*: hsCRP is a marker of systematic inflammation [25]. We categorized respondents into three groups (for males: 0 = ≤ 0.81 mg/L, 1 = 0.81–1.98 mg/L and 2 = ≥ 1.98 mg/L; for females: 0 = ≤ 0.77 mg/L, 1 = 0.77–1.86 mg/L and 2 = ≥ 1.86 mg/L) [14].

Finally, we generated the CHAI based on the sum of 6 domains and the CHAI score ranges from 0 (healthiest) to 12 (unhealthiest). To identify older adults with the ideal and the worst health, we further recoded the CHAI score as 0–2 (healthiest, also the ideal group), 3 to 4, 5 to 6, and 7–12 (the unhealthiest group). Thus our ideal healthy ageing was a dummy equal to 1 if the CHAI score ranged 0–2, 0 otherwise.

### Covariates

Sociodemographic characteristics included age, sex, marital status (married, others), education (illiterate, primary school, middle school, high school or higher). Since smoking and obesity were also two important (albeit negative) predictors for healthy ageing [16], we also included smoking and overweight (a body mass index (BMI) is 24 kg/m^2^ or above). We also added chronic diseases and social activity (1 if participating in one or more social activities, 0 otherwise). Following Wu et al. [14], we also introduced 9 regions (North, East, Central, Southwest, Northeast, Northwest, South central, Southeast and South) to capture possible geographical heterogeneity. Given the China’s great rural-urban divide, we also controlled current residence (urban and rural).

### Statistical analysis

First, we compared sample characteristics between 2011 and 2015. Then we identified the average CHAI scores and the distribution of 4 CHAI groups (0–2, 3–4, 5–6, 7–12) by sociodemographic factors, lifestyles, chronic diseases and social activity within both waves of 2011 and 2015. Subsequently, we also estimated the age-adjusted prevalence of ideal healthy ageing across 9 geographical regions in 2011 and 2015, and compared the rates of ideal CHAI over time. Next, we used ordinary least squares (OLS, model 1) and logistic model (model 2) estimates to examine the associations between sociodemographic factors and the CHAI and the prevalence of ideal CHAI, respectively.

To rule out individual-level unobserved heterogeneities, we generally used longitudinal estimates. In this study, a random effect (RE) is synonymous with zero correlation between the observed explanatory variables and individual unobserved effects. The RE here is a special version of the RE of the mixed model, in which only the intercept has distribution-based randomness. We also employed a Hausman test (under the null hypothesis, both RE and fixed effects (FE) are consistent but FE is inefficient, therefore, RE is preferable) to decide whether to use FE or RE for the longitudinal estimates. And the result for this latter indicates that the null hypothesis cannot be rejected, suggesting that the use of RE estimation is preferable in this paper. Then, we fitted RE (model 3) and RE logistic (model 4) models to explore the associations of CHAI score/the prevalence of ideal CHAI with demographic and socioeconomic characteristics, respectively. Using RE and RE logistic models allows us to rule out the potential biases associated with individual-level unobserved heterogeneity.

To identify whether various sociodemographic determinants have heterogeneous impacts on the distribution of CHAI, we estimated unconditional quantile regression (UQR) model as a simple OLS regression on a transformed dependent variable at the 25th, 50th, and 75th percentiles using the recentered influence function (RIF). The UQR approach facilitates us to understand how CHAI heterogeneously responds to changes in covariates. More importantly, UQR is a useful technique for exploring extreme impacts in the CHAI distribution, thereby providing a more comprehensive picture of the effects of the covariates on CHAI scores.

Finally, we detected the robustness of our regression using a balanced panel and adjusting for household expenditure per capita (HEPC), which was considered as a long-term measure of household economic conditions [26, 27]. We assessed the reliability of this variable using Benford test to confirm whether these data follow Benford’s law (recognizing data irregularities and number occurrences in the context of large survey data sets) [28]. With a Chi-squared statistic equal to 68.17, we clearly rejected the null hypothesis that the data follows a Benford distribution thereby raising the concern about the reliability of household expenditure data. Thus we took this variable as a robustness check rather than included in our main analysis. Given the CHARLS’s multistage probability sampling design, we adjusted for sampling weights for household and individual nonresponse and nonparticipation in blood sample collection to ensure nationally representative estimates. All analyses were conducted using Stata 16 (Stata Corp, College Station, TX).

## Results

### Sample characteristics and CHAI scores in 2011 and 2015

Table 1 showed that during the period of 2011–2015, the mean values of SBP, fasting glucose, eGFR and hsCRP declined, though with different magnitudes, whilst the average values of peak expiratory flow and TICS score significantly increased, perhaps indicating a better health profile. In addition, the overall average CHAI score was 5.42, with a decline from 5.67 in 2011 to 5.20 in 2015. Both educational categories of middle school, and high school or higher had increased by 2.0 and 3.0%, respectively. The prevalence of overweight (including obesity, BMI ≥ 24 kg/m^2^) had increased from 40.0% in 2011 to 44.0% in 2015.

**Table 1:** Characteristics of Chinese adults aged 60+ in the CHARLS 2011 and 2015 (*n* = 8182)

Variables	Total (***n*** = 8182)		2011 (***n*** = 3695)	2015 (***n*** = 4487)	MD
	Mean/percentage	Std. Dev.	Mean/percentage	Mean/percentage	
Dependent variable					
CHAI	5.42	2.08	5.67	5.20	−0.47***
SBP (mmHg)	141.50	78.15	143.15	140.02	−3.13***
Peak expiratory flow (L/min)	254.17	138.81	236.43	269.94	33.51***
Fasting glucose (mg/dL)	106.66	33.25	110.99	102.81	−8.18***
TICS score	15.72	5.81	15.54	15.89	0.35***
eGFR (mL/min per 1.73 m^2^)	76.05	11.24	76.15	75.96	−0.19***
hsCRP (mg/L)	3.17	7.53	3.39	2.98	−0.41***
Independent variables					
Age groups					
60–64	0.36	0.48	0.36	0.35	−0.01
65–69	0.27	0.44	0.25	0.28	0.03***
70–74	0.19	0.40	0.20	0.19	−0.01
75–79	0.13	0.33	0.13	0.12	−0.01
≥ 80	0.06	0.24	0.06	0.06	0.00
Sex (1 = male, 0 = female)	0.50	0.50	0.49	0.50	0.01
Marital status (1 = married, 0 = others)	0.77	0.42	0.76	0.78	0.02**
Education	1.81	0.98	1.75	1.87	0.12***
Illiterate	0.51	0.50	0.53	0.49	−0.04***
Primary school	0.25	0.44	0.26	0.25	−0.01
Middle school	0.16	0.36	0.14	0.16	0.02***
High school or higher	0.08	0.28	0.07	0.10	0.03***
Region	4.78	2.53	4.88	4.69	−0.19***
North	0.12	0.32	0.11	0.13	0.02***
East	0.06	0.24	0.07	0.06	−0.01*
Central	0.20	0.40	0.19	0.20	0.01
Southwest	0.16	0.37	0.16	0.16	0.00
Northeast	0.07	0.25	0.07	0.07	0.00
Northwest	0.07	0.26	0.09	0.06	−0.03***
South central	0.14	0.35	0.13	0.15	0.02***
South east	0.07	0.25	0.07	0.07	0.00
South	0.11	0.31	0.12	0.10	−0.02***
Current residence (1 = rural, 0 = urban)	0.56	0.50	0.56	0.56	0.00
Smoking (1 = yes, 0 = no)	0.27	0.45	0.27	0.28	0.01
Overweight or obese (BMI ≥ 24 kg/m^2^)	0.42	0.49	0.40	0.44	0.04***
Chronic disease (1 = yes, 0 = no)	0.75	0.43	0.78	0.73	−0.05***
Social activity (1 = yes, 0 = no)	0.51	0.50	0.49	0.53	0.04***

*CHARLS* China Health and Retirement Longitudinal Study, *CHAI* Chinese Healthy Ageing Index, *SBP* Systolic blood pressure, *TICS* Telephone Interview for Cognitive Status, *eGFR* Estimated glomerular filtration rate, *hsCRP* High-sensitivity C-reactive protein, *MD* Mean difference. The significance of the mean difference is based on independent t-tests. * *p* ≤ 0.1, ** *p* ≤ 0.05, *** *p* ≤ 0.01

### Sociodemographic distribution of CHAI score

Table 2 shows the distributions of CHAI scores by sociodemographic and lifestyle characteristics. Between 2011 and 2015, the proportions of the CHAI score of 0 to 2 (healthiest) had increased from 5.6 to 9.4%, whilst the score of 7 to 12 (unhealthiest) decreased from 34.4% in 2011 to 25.9% in 2015. Respondents who were younger, females, married or living together, higher education, living in urban area, non-smoking, non-overweight, non-chronic diseases and regularly participating in social activity had lower mean scores. In particular, there is a clear age-CHAI gradient, specifically, the CHAI had significantly increased from 4.651 among the age group of 60–64 to 7.340 in the age group of 80+, perhaps suggesting that an increase in age is less likely to have healthy ageing. This is also the case for both waves of 2011 and 2015 (Additional File 1 Table A1).

**Table 2:** Between group differences and over time changes in the distribution of CHAI score (ranged 0–12) in Chinese adults aged 60+ by sociodemographic characteristics: CHARLS 2011 and 2015

	CHAI (%)											
	0–2 (Healthiest)			3–4			5–6			7–12 (Unhealthiest)		
	2011	2015	Difference	2011	2015	Difference	2011	2015	Difference	2011	2015	Difference
All	5.6	9.4	3.8***	23.5	28.7	5.3***	36.6	35.9	−0.7***	34.4	25.9	−8.4***
Age groups												
60–64	10.2	15.6	5.4***	31.5	35.9	4.4***	36.8	33.3	−3.5***	21.5	15.3	−6.2***
65–69	4.8	8.6	3.8***	24.7	32.4	7.7***	40.1	37.6	−2.5***	30.4	21.5	−8.9***
70–74	1.2	4.0	2.8***	16.2	23.3	7.1***	37.9	39.2	1.3***	44.7	33.6	−11.1***
75–79	0.2	3.4	3.2***	10.6	13.9	3.3***	30.5	38.3	7.8***	58.6	44.4	−14.2***
≥ 80	0.0	0.5	0.5***	6.9	3.2	−3.7***	25.0	27.8	2.8***	68.1	68.5	0.4
Sex												
Female	6.6	11.0	4.4***	24.0	29.7	5.7***	36.0	35.1	−0.9***	33.3	24.2	−9.1***
Male	4.5	7.7	3.2***	22.9	27.8	4.9***	37.2	36.8	−0.4***	35.4	27.7	−7.7***
Marital status												
Others	2.4	6.6	4.2***	16.4	18.9	2.5***	35.7	35.3	−0.4***	45.5	39.2	−6.3***
Married	6.4	10.1	3.7***	25.2	31.2	6.0***	36.8	36.1	−0.7***	31.6	22.6	−9.0***
Education												
Illiterate	3.8	7.4	3.6***	20.1	24.8	4.7***	35.7	38.1	2.4***	40.4	29.7	−10.7***
Primary school	7.0	10.2	3.2***	26.3	29.3	3.0***	38.7	35.2	−3.5***	28.0	25.2	−2.8***
Middle school	10.2	12.4	2.2***	30.2	39.9	9.7***	36.3	30.0	−6.3***	23.3	17.7	−5.6***
High school or higher	7.6	16.4	8.8***	32.6	35.0	2.4***	37.0	33.2	−3.8***	22.8	15.4	−7.4***
Region												
North	7.8	9.1	1.3***	26.3	32.5	6.2***	36.1	35.6	−0.5**	29.8	22.9	−6.9***
East	5.8	11.4	5.6***	24.2	30.7	6.5***	38.6	32.9	−5.7***	31.4	25.0	−6.4***
Central	6.5	8.8	2.3***	20.6	31.0	10.4***	38.0	36.8	−1.2***	34.9	23.4	−11.5***
Southwest	3.2	7.1	3.9***	23.1	25.8	2.7***	36.4	36.9	0.5***	37.3	30.2	−7.1***
Northeast	6.5	11.0	4.5***	26.3	35.0	8.7***	36.0	35.0	−1.0**	31.2	18.9	−12.3***
Northwest	5.7	9.6	3.9***	26.4	24.4	−2.0***	34.1	39.9	5.8***	33.8	26.1	−7.7***
South central	4.1	11.0	6.9***	21.9	24.7	2.8***	37.5	34.1	−3.4***	36.5	30.2	−6.3***
South east	8.3	12.6	4.3***	27.8	29.6	1.8***	33.6	30.9	−2.7***	30.3	26.9	−3.4***
South	4.9	8.7	3.8***	21.7	24.7	3.0***	36.0	38.1	2.1***	37.5	28.4	−9.1***
Current residence												
Urban	5.7	10.7	5.0***	23.0	29.3	6.3***	37.1	35.0	−2.1***	34.3	25.0	−9.3***
Rural	5.5	8.7	3.2***	23.7	28.5	4.8***	36.3	36.4	0.1**	34.4	26.4	−8.0***
Smoking												
No	5.9	10.3	4.4***	24.0	28.4	4.4***	36.3	35.9	−0.4***	33.9	25.4	−8.5***
Yes	4.9	7.0	2.1***	22.2	29.8	7.6***	37.3	35.8	−1.5***	35.5	27.4	−8.1***
Overweight and obese (kg/m^2^)												
< 24	6.3	11.1	4.8***	24.2	30.1	5.9***	36.8	34.5	−2.3***	32.7	24.4	−8.3***
≥ 24	4.3	7.1	2.8***	22.2	26.9	4.7***	36.2	37.9	1.7***	37.3	28.2	−9.1***
Chronic disease												
No	8.1	11.7	3.6***	26.1	34.2	8.1***	36.3	32.8	−3.5***	29.6	21.3	−8.3***
Yes	4.8	8.5	3.7***	22.7	26.7	4.0***	36.7	37.1	0.4***	35.8	27.7	−8.1***
Social activity												
No	4.8	8.8	4.0***	21.6	27.3	5.7***	36.8	36.3	−0.5***	36.8	27.7	−9.1***
Yes	6.5	10.0	3.5***	25.6	30.2	4.6***	36.4	35.6	−0.8***	31.5	24.3	−7.2***
N	206	422		867	1290		1352	1611		1270	1164	

*CHARLS* China Health and Retirement Longitudinal Study, *CHAI* Chinese Healthy Ageing Index. * *p* ≤ 0.1, ** *p* ≤ 0.05, *** *p* ≤ 0.01

### Geographical distribution of the ideal CHAI

**Fig. 1:**
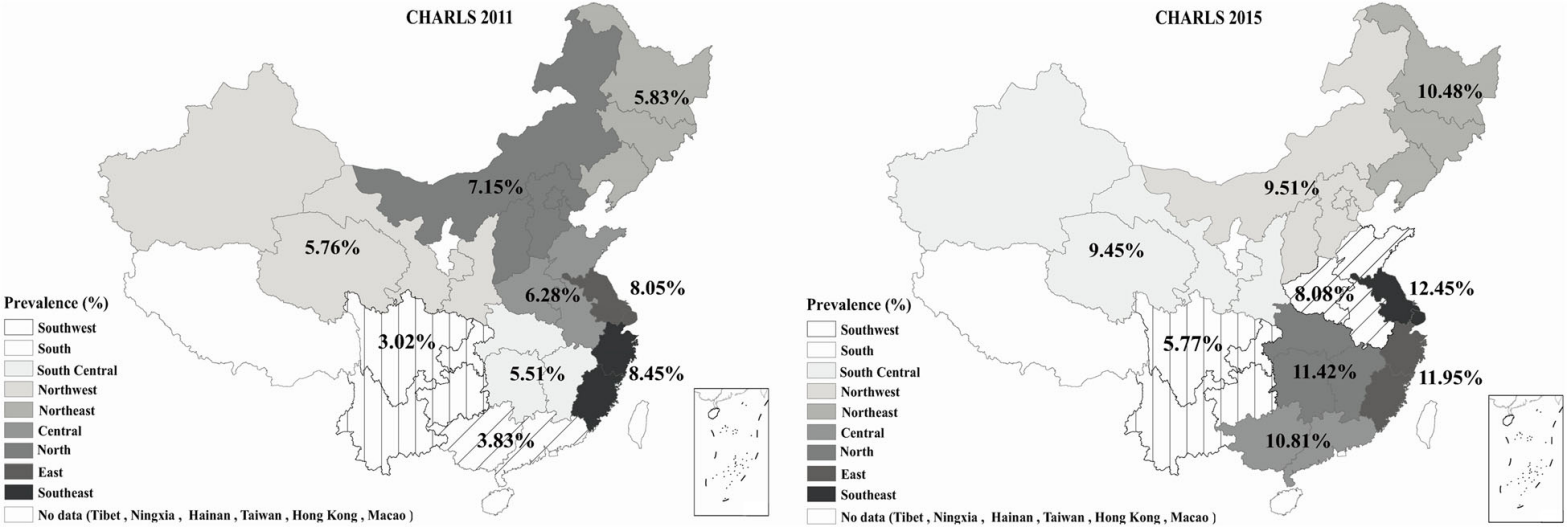
Age-adjusted distribution of the ideal Chinese Healthy Aging Index (CHAI) of adults aged 60+. The graph was drawn by authors. Weighted percentage of ideal CHAI (score: 0–2) was estimated at the weighted mean age in each district of CHARLS

**Fig. 2:**
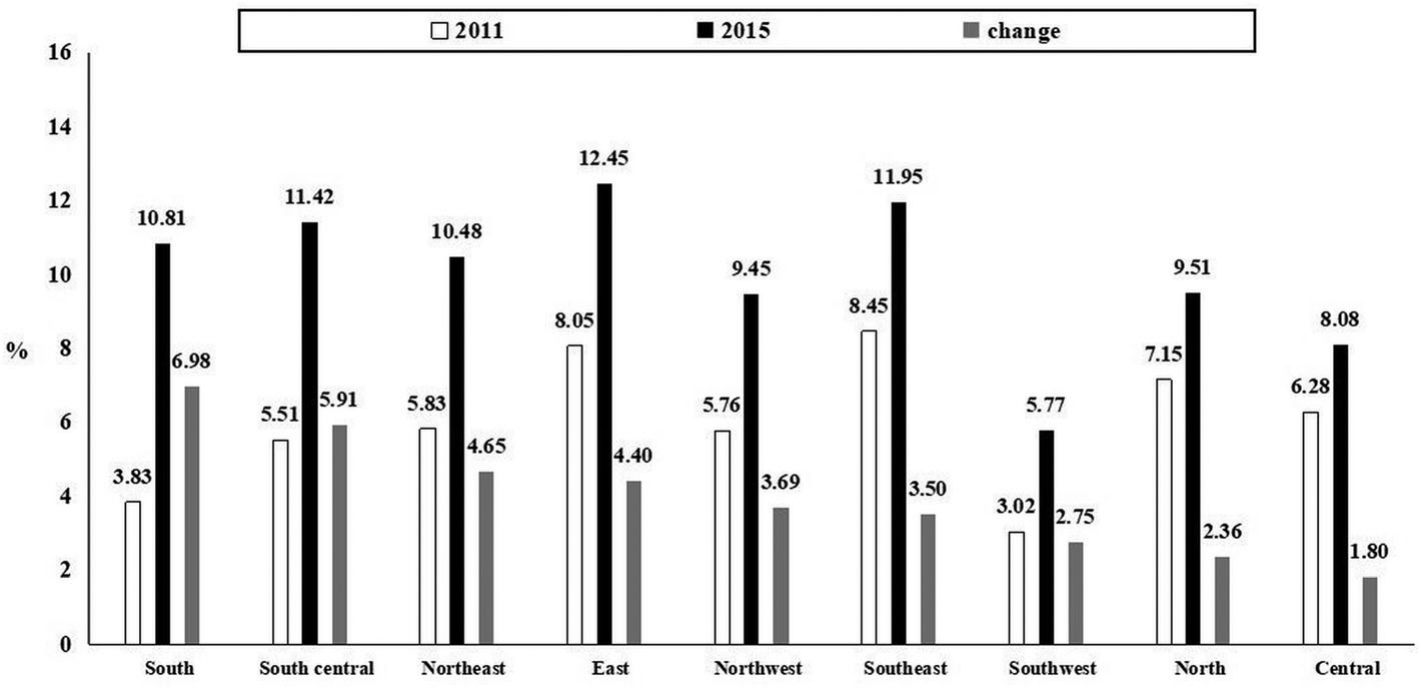
Change and geographical differences in age-adjusted distribution of the ideal Chinese Healthy Aging Index (CHAI) of adults aged 60+. Weighted percentage of ideal CHAI (score: 0–2) was estimated at the weighted mean age in each district of CHARLS. The figure is arranged according to the change (2015 minus 2011) in the weighted percentage of ideal CHAI (score: 0–2) between 2011 and 2015

Figure 1 illustrates geographical distributions of ideal CHAI (score 0–2) prevalence in waves of 2011 and 2015. The age-adjusted rates of the ideal CHAI differed substantially according to geographical region, ranging from 3.0% in the Southwest to 8.5% in the Southeast in 2011 (in 2015, ranging from 5.8% in the Southwest to 12.5% in the East). Also, the top three regions with the greatest improvements in the prevalence of the ideal CHAI included the South, South Central and Northeast (Fig. 2).

### Sociodemographic predictors of CHAI

As indicated in Table 3, the older aged 65+, male, living in the Center and West areas, smoking, overweight and having chronic diseases were positively associated with CHAI score (Models 1 and 2). However, being married/living together, having higher education and regularly participating in social activity were negatively correlated with the CHAI score. Relative to the wave of 2011, coefficients of the 2015 wave was significantly negative (Model 1: -0.41, 95% CIs = [− 0.52, − 0.29]; Model 2: -0.43, 95% CIs = [− 0.50, − 0.36]). Similarly, those 65 years old and over, being male, living in the West, smoking, being obese/overweight and suffering from chronic diseases were less likely to have an ideal CHAI profile, whilst those who were married or living together, more educated and regularly participating in activity were more prone to have an ideal CHAI (Models 3 and 4).

**Table 3:** OLS/logistic/RE/RE logistic estimates for sociodemographic determinants of CHAI score and the prevalence of ideal CHAI, CHARLS 2011 and 2015

Variables	CHAI score Coefficients (95% CIs)		Prevalence of ideal CHAI (CHAI score: 0–2) Odds Ratio (95% CIs)	
	OLS	RE	Logistic	RE logistic
	Model 1	Model 2	Model 3	Model 4
**Age groups**				
60–64 (ref.)				
65–69	0.50*** (0.36, 0.63)	0.51*** (0.41, 0.59)	0.49*** (0.38, 0.64)	0.41*** (0.31, 0.52)
70–74	1.16*** (0.97, 1.35)	1.24*** (1.13, 1.35)	0.30*** (0.13, 0.72)	0.12*** (0.08, 0.19)
75–79	1.81*** (1.63, 1.99)	1.71*** (1.57, 1.85)	0.14*** (0.07, 0.27)	0.09*** (0.05, 0.17)
≥ 80	2.44*** (2.15, 2.73)	2.42*** (2.22, 2.62)	0.01*** (0.00, 0.06)	0.01*** (0.00, 0.08)
**Sex**				
Female (ref.)				
Male	0.50*** (0.35, 0.64)	0.48*** (0.37, 0.58)	0.45*** (0.32, 0.63)	0.46*** (0.34, 0.61)
**Marital status**				
Others (ref.)				
Married	−0.37*** (− 0.52, − 0.23)	− 0.32*** (− 0.43, − 0.21)	1.31 (0.90, 1.89)	1.34* (0.96, 1.85)
**Education**				
Illiterate (ref.)				
Primary school	− 0.41*** (− 0.55, − 0.28)	−0.46*** (− 0.57, − 0.35)	1.88*** (1.47, 2.40)	2.25*** (1.69, 3.02)
Middle school	−1.06*** (−1.29, − 0.83)	−0.80*** (− 0.94, − 0.66)	3.29*** (2.07, 5.25)	2.79*** (1.97, 3.97)
High school or higher	−1.03*** (− 1.26, − 0.81)	−1.03*** (− 1.23, − 0.83)	2.79*** (1.79, 4.34)	4.08*** (2.53, 6.58)
**Region**				
North (ref.)				
East	− 0.08 (− 0.36, 0.19)	−0.19*** (− 0.42, 0.04)	1.31 (0.74, 2.31)	1.52 (0.87, 2.64)
Central	0.15 (−0.04, 0.33)	0.03 (−0.12, 0.19)	0.99 (0.68, 1.42)	1.07 (0.73, 1.56)
Southwest	0.35*** (0.14, 0.56)	0.25*** (0.09, 0.41)	0.64** (0.42, 0.99)	0.69* (0.45, 1.06)
Northeast	0.21* (−0.03, 0.45)	0.04 (−0.18, 0.25)	0.77 (0.46, 1.30)	0.92 (0.55, 1.55)
Northwest	0.42*** (0.17, 0.68)	0.19* (−0.02, 0.39)	0.92 (0.56, 1.50)	0.98 (0.59, 1.63)
South central	0.36*** (0.15, 0.57)	0.19** (0.02, 0.36)	1.03 (0.68, 1.57)	1.04 (0.68, 1.60)
South east	0.06 (−0.22, 0.35)	−0.18 (− 0.39, 0.03)	1.61* (0.97, 2.67)	1.70** (1.03, 2.81)
South	0.04 (−0.33, 0.40)	0.26*** (0.05, 0.46)	0.97 (0.37, 2.54)	0.73 (0.43, 1.24)
**Current residence**				
Urban (ref.)				
Rural	0.03 (−0.09, 0.15)	−0.02 (− 0.12, 0.08)	0.93 (0.75, 1.15)	0.91 (0.71, 1.17)
**Smoking**				
No (ref.)				
Yes	0.18** (0.04, 0.31)	0.12** (0.02, 0.23)	0.76* (0.57, 1.03)	0.69*** (0.50, 0.90)
**Weight status**				
BMI < 24 (ref.)				
BMI ≥ 24 (overweight)	0.64*** (0.52, 0.76)	0.60*** (0.51, 0.69)	0.46*** (0.33, 0.64)	0.37*** (0.28, 0.47)
**Chronic disease**				
No (ref.)				
Yes	0.26*** (0.14, 0.38)	0.35*** (0.25, 0.45)	0.77** (0.59, 1.00)	0.67*** (0.52, 0.85)
**Social activity**				
No (ref.)				
Yes	−0.21*** (− 0.32, − 0.10)	− 0.17*** (− 0.24, − 0.09)	1.12* (0.90, 1.41)	1.21* (0.97, 1.51)
**Survey year**				
2011 (ref.)				
2015	− 0.41*** (− 0.52, − 0.29)	− 0.43*** (− 0.50, − 0.36)	1.89*** (1.46, 2.44)	2.08*** (1.66, 2.60)
**N**	8182	8182	8182	8182
***Adj.R***^***2***^***/Pseudo R***^***2***^	0.25	0.24	0.14	

*CHARLS* China Health and Retirement Longitudinal Study, *CHAI* Chinese Healthy Ageing Index, *OLS* Ordinary least squares, *RE* Random effects. * *p* ≤ 0.1, ** *p* ≤ 0.05, *** *p* ≤ 0.01

Compared with the wave of 2011, those respondents in the 2015 wave were likely to have an ideal CHAI (Model 3: odds ratio (OR) = 1.89, 95% CIs = [1.46, 2.44]; Model 4: OR = 2.08, 95% CIs = [1.66, 2.60]). After adjusting for HEPC, we observed similar results to those without controlling for HEPC (Additional File 2 Table A2). We still found similar results when using a balanced panel (Additional File 3 Table A3) to those in Table 3 when using an unbalanced panel.

### Heterogeneous impacts of sociodemographic determinants of CHAI

Table 4 shows heterogeneous impacts of sociodemographic characteristics on the distribution of CHAI. We found that those in the upper part of the distribution (75th percentile) were more sensitive to the positive predicators (older age, male, living in the Center and West, smoking, overweight and having chronic diseases) of the CHAI scores than those in the median and lower part of the distributions (50th and 25th percentiles) with the exception of sex. However, regarding the negative predictors (married/living together, more educated, participating in social activity and in the wave of 2015) of the CHAI scores, we did not observe any stronger effects at the upper tail of the distribution of CHAI scores except for the follow-up wave.

**Table 4:** Unconditional quantile regression for sociodemographic determinants of CHAI score among older adults aged 60+, CHARLS 2011 and 2015

Variables	25th Coefficients (95% CIs)	50th Coefficients (95% CIs)	75th Coefficients (95% CIs)
CHAI score	4.34	5.78	7.34
**Age groups**			
60–64 (ref.)			
65–69	0.49*** (0.22, 0.75)	0.49*** (0.29, 0.68)	0.40*** (0.23, 0.57)
70–74	0.96*** (0.72, 1.20)	1.09*** (0.90, 1.28)	1.10*** (0.85, 1.35)
75–79	1.21*** (1.01, 1.41)	1.50*** (1.31, 1.68)	2.11*** (1.81, 2.42)
≥ 80	1.41*** (1.20, 1.62)	1.86*** (1.67, 2.06)	2.92*** (2.49, 3.34)
**Sex**			
Female (ref.)			
Male	0.55*** (0.35, 0.76)	0.53*** (0.37, 0.69)	0.38*** (0.17, 0.58)
**Marital status**			
Others (ref.)			
Married	−0.30*** (− 0.46, − 0.14)	− 0.40*** (− 0.55, − 0.25)	− 0.32*** (− 0.52, − 0.11)
**Education**			
Illiterate (ref.)			
Primary school	− 0.42*** (− 0.57, − 0.27)	− 0.39*** (− 0.53, − 0.25)	− 0.42*** (− 0.62, − 0.22)
Middle school	−1.08*** (−1.50, − 0.67)	− 1.17*** (− 1.45, − 0.88)	− 0.85*** (− 1.09, − 0.60)
High school or higher	−1.08*** (− 1.46, − 0.71)	−1.04*** (− 1.33, − 0.75)	− 0.97*** (− 1.30, − 0.64)
**Region**			
North (ref.)			
East	− 0.18 (− 0.54, 0.19)	− 0.06 (− 0.37, 0.25)	−0.01 (− 0.39, 0.36)
Central	0.10 (− 0.14, 0.34)	0.12 (− 0.09, 0.34)	0.19 (− 0.08, 0.45)
Southwest	0.22* (− 0.03, 0.48)	0.24** (0.01, 0.47)	0.47*** (0.19, 0.76)
Northeast	0.26 (−0.06, 0.59)	0.21 (−0.08, 0.50)	0.25 (− 0.10, 0.61)
Northwest	0.29** (0.01, 0.57)	0.32** (0.06, 0.58)	0.59*** (0.22, 0.95)
South central	0.24* (−0.02, 0.50)	0.29** (0.05, 0.52)	0.51*** (0.21, 0.80)
South east	−0.16 (− 0.48, 0.16)	−0.17 (− 0.46, 0.12)	0.35* (− 0.03, 0.73)
South	−0.23 (− 0.87, 0.41)	0.01 (− 0.41, 0.44)	0.19 (− 0.27, 0.65)
**Current residence**			
Urban (ref.)			
Rural	0.06 (− 0.07, 0.19)	−0.02 (− 0.14, 0.10)	0.01 (− 0.17, 0.18)
**Smoking**			
No (ref.)			
Yes	0.08 (−0.09, 0.25)	0.10 (−0.05, 0.25)	0.25** (0.05, 0.46)
**Weight status**			
BMI < 24 (ref.)			
BMI ≥ 24 (overweight)	0.56*** (0.39, 0.73)	0.58*** (0.45, 0.72)	0.65*** (0.47, 0.83)
**Chronic disease**			
No (ref.)			
Yes	0.24*** (0.07, 0.42)	0.29*** (0.15, 0.43)	0.25*** (0.09, 0.41)
**Social activity**			
No (ref.)			
Yes	−0.19*** (−0.33, −0.05)	−0.22*** (− 0.34, − 0.10)	−0.26*** (− 0.42, − 0.09)
**Survey year**			
2011 (ref.)			
2015	−0.36*** (− 0.53, − 0.18)	−0.38*** (− 0.51, − 0.24)	−0.36*** (− 0.52, − 0.20)
**N**	8182	8182	8182
***Adj.R***^***2***^	0.12	0.17	0.16

*CHARLS* China Health and Retirement Longitudinal Study, *CHAI* Chinese Healthy Ageing Index. * *p* ≤ 0.1, ** *p* ≤ 0.05, *** *p* ≤ 0.01

## Discussion

For the first time, using the unique large nationally representative survey data, we constructed the CHAI, tracked its changes, and assessed its sociodemographic determinants. We found that the mean CHAI score decreased from 5.67 in 2011 to 5.20 in 2015. The proportion of subjects that had a CHAI score of 0 to 2 (healthiest) increased from 5.6 to 9.4%. Substantial heterogeneity in CHAI score by different social and spatial characteristics existed. The age-adjusted rates of the ideal CHAI ranged from 3.0% in the Southwest to 8.5% in the Southeast in 2011 (in 2015, ranging from 5.8% in the Southwest to 12.5% in the East).

The rate of having an ideal CHAI profile also varied substantially according to nine geographic regions. The huge heterogeneity also existed in the improvements of prevalence of having an ideal CHAI profile during this five-year period, with the top three regions including South, South Central and Northeast. We also found that respondents who were 65 years old and over, male, living in the West, smoking, overweight and suffering from chronic diseases were positively associated with CHAI score and less likely to have an ideal CHAI profile (score 0–2), but those who were married/living together, more educated and regularly participating in activity were negatively linked with CHAI score and more prone to have an ideal CHAI profile. And those in the upper part of the CHAI distribution (75th percentile) were more sensitive to the positive predicators of the CHAI scores than those in the median and lower part (25th percentile) of the distributions except for sex.

Our findings were consistent with previous findings based on the 2011 CHARLS data, indicating respondents who were younger, female, more educated and married/living together had lower mean scores of CHAI and more likely to have the healthiest CHAI score [7, 10, 11]. Our findings were similar as those in the U.S., where the younger, females and high educated were more prone to have an ideal HAI profile. Geographical heterogeneity in socioeconomic conditions [29], unbalanced distribution of social health insurance [30], and environmental conditions [31] may partially explain spatial differences in healthy ageing. And such sizable geographical variations in the prevalence of the ideal CHAI may help account for China’s regional inequalities in health and healthcare expenditures [31–33].

Heterogeneous impacts of sociodemographic determinants of healthy ageing, in particular, stronger effects at the upper distribution of CHAI score, implies that the CHAI would be valuable in identifying those at the very high risk of outcomes associated with sociodemographic characteristics. In addition, our results also confirmed that high education produced a higher probability of the ideal CHAI, which may underscore the importance of education in healthy ageing promotion. One possibility is that higher education would help individuals become more health-conscious and take preventive actions. Existing studies in China also showed that education is the most important predictor for the improvement in cognitive health of Chinese [16, 27]. Moreover, our findings were in accordance with these existing studies on obesity, health-related behaviors and healthy ageing/longevity, confirming that smoking and overweight were detrimental to healthy ageing [16, 33–37].

CHAI is a useful tool to identify older adults at risk of adverse outcomes and also provides a complementary explanation of the variation in health outcomes [14]. Future research may test the application of such index scores in policy making in relation to health and wellbeing of older people.

Our study has three limitations. First, as in most longitudinal studies, selective attrition may exist. Specifically, healthy respondents are more likely to remain in the study during follow-ups and those with (severe) health problems are more likely to drop-out. Respondents in this study may represent a slightly healthier part of the population. Second, we assessed the changes of the CHAI within a relatively short time period (2011 to 2015). Future research may, when more waves of data available, track long-term dynamics of the CHAI in Chinese older adults. Third, our results may depend in part on the construction of the CHAI, in particular, the included measures and cutoffs used to clearly define a three-point scale of each measure. Due to the tentative nature of the CHAI, more studies are needed to examine the scientific validity or practical value of the CHAI. Although the choice of cutoffs was arbitrary, the best score of 0 was generally found to represent a healthy, young normal value, and value of 2 were in the range of individuals with diagnosed chronic disease [6].

This study has a few key strengths. First, in the CHARLS, the response rate of the tracked sample (panel sample) is higher than 86% in any of the follow-up waves. In 2015, 87.15% of the baseline households were tracked. The success rates are much higher compared with many HRS-type surveys [38]. Second, it filled a gap in the literature on the change of healthy ageing and its potential sociodemographic determinants in a developing context. Using a large-scale nationally representative sample would facilitate the possible generalization of our findings. Third, the six domains of CHAI were selected from noninvasive measure of organ structure and function, thereby allowing for quantification of a broad range of capacity from severely diseased or diminished to robust and healthy [11]. The use of noninvasive measures that yield continuous measures of age and disease-associated changes facilitate us to provide a complementary explanation of the variation in risk of outcome and to identify older adults with exceptional health [14]. Finally, we detected heterogeneous response of sociodemographic characteristics on the whole distribution of CHAI score using an UQR technique, highlighting the importance of the use of “beyond the mean” approach in exploring demographic and socioeconomic determinants of healthy ageing.

Our findings have some policy and public health implications. First, substantial geographical and sociodemographic heterogeneities in the prevalence of the ideal CHAI underscore health inequality in old ages remains a challenge in China, though overall level of health outcome has improved substantially [33]. Thus, reducing health inequalities at the subnational level would need targeted and tailored policies or interventions to improve major socioeconomic factors (e.g. education) and lessen disease burden in the regional level. Second, various strategies for tobacco control have not been effectively implemented in the past decade or more and the prevalence of smoking has not substantially declined among older adults (actually a slight increase during 2011–2015 period). Thus more efforts are needed to put into practice the call for integrating health into all relevant policies. Finally, given the sharp increase in obesity/overweight rates among the study population and its detrimental effects on healthy ageing, obesity control and prevention strategies, policies, actions and plans, such as “Healthy China Action (2019-2030)”, would motivate the whole societies against non-communicable diseases. In particular, China‘s “*Basic Healthcare and Health Promotion Law*” (enacted on June 1, 2020) [39] would integrate health education into the national education system and carry out nutrition improvement interventions for older adults, promote their healthy eating habits and reduce disease risk.

## Conclusion

In conclusion, this study advances our understanding of health ageing in a less developed setting, especially the change of health ageing and its potential sociodemographic determinants. Assessing potential sociodemographic and lifestyle determinants of older adults with exceptional health over time may be valuable for minimizing disease burden and promoting healthy life in Chinese older adults. Future policies and interventions should especially focus on men, those in Central and West China, and combat health problems like obesity, chronic diseases and unhealthy behaviors.

## Supplementary Information

**Additional file 1: Table A1.** CHAI by age groups.

**Additional file 2: Table A2.** OLS/logistic/RE estimates for sociodemographic determinants of CHAI score and the prevalence of ideal CHAI: CHARLS 2011 and 2015 (unbalanced panel with household expenditure as an additional covariate, *n* = 6141). CHARLS = China Health and Retirement Longitudinal Study. CHAI=Chinese Healthy Ageing Index. OLS = ordinary least squares. RE = random effects. * *p* ≤ 0.1, ** *p* ≤ 0.05, *** *p* ≤ 0.01.

**Additional file 3: Table A3.** OLS/logistic/RE estimates for sociodemographic determinants of CHAI score and the prevalence of ideal CHAI: CHARLS 2011 and 2015 (balanced panel, *n* = 3882). CHARLS = China Health and Retirement Longitudinal Study. CHAI=Chinese Healthy Ageing Index. OLS = ordinary least squares. RE = random effects. * *p* ≤ 0.1, ** *p* ≤ 0.05, *** *p* ≤ 0.01.

## Data Availability

The datasets used and analysed during the current study are available in http://forum.charls.pku.edu.cn/.

## Abbreviations

BMI: Body mass index
CHAI: Chinese Healthy Ageing Index
CHARLS: China Health and Retirement Longitudinal Study
eGFR: Estimated glomerular filtration rate
HAI: Healthy Ageing Index
hsCRP: High-sensitivity C-reactive protein
HEPC: Household expenditure per capita
OR: Odds ratio
RIF: Recentered influence function
SDGs: Sustainable Development Goals
SBP: Systolic blood pressure
TICS: Telephone Interview for Cognitive Status
UQR: Unconditional quantile regression
