# Supplementary material for: The change and correlates of healthy ageing among Chinese older adults: findings from the China health and retirement longitudinal study
Source: BMC Geriatr. 2021 Jan 27;21:78. doi: 10.1186/s12877-021-02026-y (PMC7839192; doi:10.1186/s12877-021-02026-y)
Supplement: Supplementary file 1 — Additional file 1: Table A1. CHAI by age groups. [file 12877_2021_2026_MOESM1_ESM.docx]

Table A1 CHAI by age groups

|  | **CHAI** | | | | |
| --- | --- | --- | --- | --- | --- |
|  | **60-64** | **65-69** | **70-74** | **75-79**  **75-79** | **80+** |
| All | 4.651 | 5.152 | 5.825 | 6.627 | 7.340 |
| 2011 | 4.845 | 5.382 | 6.197 | 6.956 | 7.343 |
| 2015 | 4.516 | 4.995 | 5.546 | 6.365 | 7.337 |

CHAI=Chinese Healthy Ageing Index.
