# Supplementary material for: The change and correlates of healthy ageing among Chinese older adults: findings from the China health and retirement longitudinal study
Source: BMC Geriatr. 2021 Jan 27;21:78. doi: 10.1186/s12877-021-02026-y (PMC7839192; doi:10.1186/s12877-021-02026-y)
Supplement: Supplementary file 2 — Additional file 2: Table A2. OLS/logistic/RE estimates for sociodemographic determinants of CHAI score and the prevalence of ideal CHAI: CHARLS 2011 and 2015 (unbalanced panel with household expenditure as an additional covariate, n = 6141). CHARLS = China Health and Retirement Longitudinal Study. CHAI=Chinese Healthy Ageing Index. OLS = ordinary least squares. RE = random effects. * p ≤ 0.1, ** p ≤ 0.05, *** p ≤ 0.01. [file 12877_2021_2026_MOESM2_ESM.docx]

**Table A2** OLS/logistic/RE estimates for sociodemographic determinants of CHAI score and the prevalence of ideal CHAI: CHARLS 2011 and 2015 (unbalanced panel with household expenditure as an additional covariate, n = 6,141)

| **Variables** | **CHAI score**  Coefficients (95% CIs) | |  | **Prevalence of ideal CHAI**  Odds Ratio (95% CIs) | |
| --- | --- | --- | --- | --- | --- |
|  | **OLS**  **Model 1** | **RE**  **Model 2** |  | **Logistic**  **Model 3** | **RE logistic**  **Model 4** |
| **Age groups** |  |  |  |  |  |
| 60-64 (ref.) |  |  |  |  |  |
| 65-69 | 0.47*** | 0.48*** |  | 0.56*** | 0.46*** |
|  | (0.31, 0.62) | (0.38, 0.59) |  | (0.41, 0.76) | (0.34, 0.61) |
| 70-74 | 1.07*** | 1.19*** |  | 0.39** | 0.14*** |
|  | (0.86, 1.29) | (1.06, 1.32) |  | (0.16, 0.99) | (0.09, 0.24) |
| 75-79 | 1.74*** | 1.64*** |  | 0.16*** | 0.09*** |
|  | (1.53, 1.94) | (1.47, 1.80) |  | (0.07, 0.36) | (0.04, 0.20) |
| ≥80 | 2.32*** | 2.30*** |  | 0.02*** | 0.02*** |
|  | (1.94, 2.69) | (2.06, 2.54) |  | (0.00, 0.13) | (0.00, 0.17) |
| **Sex** |  |  |  |  |  |
| Female (ref.) |  |  |  |  |  |
| Male | 0.47*** | 0.43*** |  | 0.47*** | 0.47*** |
|  | (0.30, 0.63) | (0.31, 0.55) |  | (0.32, 0.71) | (0.34, 0.66) |
| **Marital status** |  |  |  |  |  |
| Others (ref.) |  |  |  |  |  |
| Married | -0.27*** | -0.23*** |  | 1.46* | 1.36 |
|  | (-0.43, -0.11) | (-0.36, -0.10) |  | (0.95, 2.24) | (0.91, 2.02) |
| **Education** |  |  |  |  |  |
| Illiterate (ref.) |  |  |  |  |  |
| Primary school | -0.40*** | -0.45*** |  | 1.99*** | 2.56*** |
|  | (-0.55, -0.25) | (-0.58, -0.33) |  | (1.50, 2.64) | (1.83, 3.59) |
| Middle school | -1.01*** | -0.76*** |  | 3.05*** | 2.67*** |
|  | (-1.25, -0.77) | (-0.92, -0.60) |  | (1.85, 5.05) | (1.78, 4.00) |
| High school or higher | -0.84*** | -0.96*** |  | 2.00** | 3.66*** |
|  | (-1.11, -0.58) | (-1.18, -0.73) |  | (1.13, 3.53) | (2.11, 6.33) |
| **Region** |  |  |  |  |  |
| North (ref.) |  |  |  |  |  |
| East | -0.12 | -0.29** |  | 1.25 | 1.79* |
|  | (-0.42, 0.18) | (-0.55, -0.04) |  | (0.67, 2.35) | (0.98, 3.30) |
| Central | 0.13 | 0.03 |  | 0.94 | 0.99 |
|  | (-0.08, 0.35) | (-0.14, 0.21) |  | (0.63, 1.42) | (0.65, 1.52) |
| Southwest | 0.38*** | 0.28*** |  | 0.60** | 0.61** |
|  | (0.14, 0.61) | (0.10, 0.46) |  | (0.37, 0.98) | (0.37, 0.99) |
| Northeast | 0.23* | 0.01 |  | 0.72 | 0.90 |
|  | (-0.03, 0.49) | (-0.23, 0.23) |  | (0.42, 1.24) | (0.51, 1.57) |
| Northwest | 0.46*** | 0.22* |  | 0.82 | 0.85 |
|  | (0.17, 0.76) | (-0.02, 0.45) |  | (0.45, 1.47) | (0.47, 1.54) |
| South central | 0.35*** | 0.20** |  | 0.96 | 0.89 |
|  | (0.11, 0.60) | (0.00, 0.39) |  | (0.60, 1.53) | (0.54, 1.45) |
| South east | 0.07 | -0.09 |  | 1.28 | 1.52 |
|  | (-0.23, 0.37) | (-0.34, 0.16) |  | (0.72, 2.26) | (0.83, 2.77) |
| South | 0.01 | 0.25*** |  | 0.94 | 0.59 |
|  | (-0.43, 0.42) | (0.01, 0.49) |  | (0.31, 2.80) | (0.31, 1.14) |
| **Current residence** |  |  |  |  |  |
| Urban (ref.) |  |  |  |  |  |
| Rural | -0.02 | -0.06 |  | 1.07 | 0.99 |
|  | (-0.16, 0.11) | (-0.17, 0.05) |  | (0.83, 1.39) | (0.75, 1.32) |
| **Smoking** |  |  |  |  |  |
| No (ref.) |  |  |  |  |  |
| Yes | 0.18** | 0.14** |  | 0.83 | 0.77 |
|  | (0.02, 0.33) | (0.03, 0.26) |  | (0.59, 1.15) | (0.56, 1.08) |
| **Weight status** |  |  |  |  |  |
| BMI<24 (ref.) |  |  |  |  |  |
| BMI≥24 (overweight) | 0.58*** | 0.57*** |  | 0.49*** | 0.37*** |
|  | (0.45, 0.72) | (0.47, 0.67) |  | (0.34, 0.70) | (0.28, 0.51) |
| **Chronic disease** |  |  |  |  |  |
| No (ref.) |  |  |  |  |  |
| Yes | 0.26*** | 0.38*** |  | 0.69*** | 0.57*** |
|  | (0.13, 0.40) | (0.27, 0.50) |  | (0.51, 0.93) | (0.43, 0.76) |
| **Social activity** |  |  |  |  |  |
| No (ref.) |  |  |  |  |  |
| Yes | -0.20*** | -0.17*** |  | 1.09 | 1.24* |
|  | (-0.33, -0.07) | (-0.26, -0.08) |  | (0.84, 1.41) | (0.96, 1.59) |
| **HH expenditure per capita** | -0.14*** | -0.06** |  | 1.20* | 1.09 |
|  | (-0.21, -0.06) | (-0.11, -0.01) |  | (0.98, 1.46) | (0.94, 1.26) |
| **Survey year** |  |  |  |  |  |
| 2011 (ref.) |  |  |  |  |  |
| 2015 | -0.39*** | -0.45*** |  | 1.73*** | 2.07*** |
|  | (-0.53, -0.25) | (-0.53, -0.36) |  | (1.30, 2.31) | (1.58, 2.72) |
| **N** | 6141 | 6141 |  | 6141 | 6141 |
| ***Adj.R^2^/Pseudo R^2^*** | 0.24 | 0.22 |  | 0.12 |  |

CHARLS= China Health and Retirement Longitudinal Study. CHAI=Chinese Healthy Ageing Index. OLS=ordinary least squares. RE=random effects. * p≤0.1, ** p≤0.05, *** p≤0.01.
