# Supplementary material for: The change and correlates of healthy ageing among Chinese older adults: findings from the China health and retirement longitudinal study
Source: BMC Geriatr. 2021 Jan 27;21:78. doi: 10.1186/s12877-021-02026-y (PMC7839192; doi:10.1186/s12877-021-02026-y)
Supplement: Supplementary file 3 — Additional file 3: Table A3. OLS/logistic/RE estimates for sociodemographic determinants of CHAI score and the prevalence of ideal CHAI: CHARLS 2011 and 2015 (balanced panel, n = 3882). CHARLS = China Health and Retirement Longitudinal Study. CHAI=Chinese Healthy Ageing Index. OLS = ordinary least squares. RE = random effects. * p ≤ 0.1, ** p ≤ 0.05, *** p ≤ 0.01. [file 12877_2021_2026_MOESM3_ESM.docx]

**Table A3** OLS/logistic/RE estimates for sociodemographic determinants of CHAI score and the prevalence of ideal CHAI: CHARLS 2011 and 2015 (balanced panel, n = 3,882)

| **Variables** | **CHAI score**  Coefficients (95% CIs) | |  | **Prevalence of ideal CHAI**  Odds Ratio (95% CIs) | |
| --- | --- | --- | --- | --- | --- |
|  | **OLS** | **RE** |  | **Logistic** | **RE logistic** |
| **Age groups** |  |  |  |  |  |
| 60-64 (ref.) |  |  |  |  |  |
| 65-69 | 0.32*** | 0.36*** |  | 0.50*** | 0.48*** |
|  | (0.13, 0.52) | (0.23, 0.50) |  | (0.31, 0.82) | (0.33, 0.71) |
| 70-74 | 0.92*** | 1.00*** |  | 0.41* | 0.16*** |
|  | (0.67, 1.17) | (0.82, 1.17) |  | (0.16, 1.03) | (0.09, 0.29) |
| 75-79 | 1.64*** | 1.53*** |  | 0.11*** | 0.06*** |
|  | (1.39, 1.90) | (1.30, 1.76) |  | (0.04, 0.36) | (0.02, 0.16) |
| ≥80 | 2.40*** | 2.19*** |  | 1.00 | 1.00 |
|  | (2.04, 2.76) | (1.86, 2.52) |  |  |  |
| **Sex** |  |  |  |  |  |
| Female (ref.) |  |  |  |  |  |
| Male | 0.37*** | 0.35*** |  | 0.45** | 0.71 |
|  | (0.16, 0.58) | (0.19, 0.52) |  | (0.24, 0.86) | (0.46, 1.09) |
| **Marital status** |  |  |  |  |  |
| Others (ref.) |  |  |  |  |  |
| Married | -0.44*** | -0.41*** |  | 1.82* | 1.63* |
|  | (-0.63, -0.25) | (-0.58, -0.25) |  | (0.97, 3.42) | (0.95, 2.80) |
| **Education** |  |  |  |  |  |
| Illiterate (ref.) |  |  |  |  |  |
| Primary school | -0.47*** | -0.40*** |  | 2.00*** | 2.09*** |
|  | (-0.63, -0.30) | (-0.57, -0.24) |  | (1.37, 2.90) | (1.35, 3.25) |
| Middle school | -1.22*** | -0.87*** |  | 4.12*** | 2.59*** |
|  | (-1.54, -0.90) | (-0.10, -0.64) |  | (2.02, 8.40) | (1.49, 4.50) |
| High school or higher | -1.00*** | -0.96*** |  | 2.99*** | 2.65** |
|  | (-1.32, -0.68) | (-1.31, -0.60) |  | (1.46, 6.16) | (1.16, 6.04) |
| **Region** |  |  |  |  |  |
| North (ref.) |  |  |  |  |  |
| East | -0.20 | -0.24 |  | 2.38* | 3.07** |
|  | (-0.62, -0.23) | (-0.62, 0.14) |  | (1.00, 5.66) | (1.25, 7.53) |
| Central | 0.01 | -0.02 |  | 1.34 | 1.56 |
|  | (-0.22, 0.25) | (-0.27, 0.23) |  | (0.71, 2.50) | (0.84, 2.91) |
| Southwest | 0.21 | 0.11 |  | 0.50* | 0.57 |
|  | (-0.06, 0.48) | (-0.16, 0.38) |  | (0.23, 1.07) | (0.27, 1.20) |
| Northeast | 0.11 | -0.20 |  | 0.84 | 1.48 |
|  | (-0.23, 0.45) | (-0.55, 0.16) |  | (0.37, 1.88) | (0.64, 3.41) |
| Northwest | 0.19 | 0.06 |  | 0.70 | 0.71 |
|  | (-0.10, 0.49) | (-0.26, 0.38) |  | (0.29, 1.65) | (0.30, 1.70) |
| South central | 0.39** | 0.30** |  | 0.83 | 0.88 |
|  | (0.12, 0.66) | (0.02, 0.58) |  | (0.40, 1.72) | (0.42, 1.81) |
| South east | -0.08 | -0.18 |  | 1.92* | 2.07* |
|  | (-0.40, 0.24) | (-0.51, 0.15) |  | (0.92, 3.98) | (0.94 , 4.56) |
| South | -0.40 | 0.19 |  | 1.40 | 0.55 |
|  | (-0.92, 0.12) | (-0.18, 0.55) |  | (0.32, 6.05) | (0.19, 1.59) |
| **Current residence** |  |  |  |  |  |
| Urban (ref.) |  |  |  |  |  |
| Rural | -0.03 | -0.13 |  | 0.99 | 0.95 |
|  | (-0.19, 0.12) | (-0.29, 0.03) |  | (0.70, 1.40) | (0.63, 1.42) |
| **Smoking** |  |  |  |  |  |
| No (ref.) |  |  |  |  |  |
| Yes | 0.27*** | 0.14* |  | 0.88 | 0.60** |
|  | (0.09, 0.45) | (-0.02, 0.29) |  | (0.55, 1.39) | (0.38, 0.94) |
| **Weight status** |  |  |  |  |  |
| BMI<24 (ref.) |  |  |  |  |  |
| BMI≥24 (overweight) | 0.71*** | 0.58*** |  | 0.39*** | 0.32*** |
|  | (0.55, 0.86) | (0.44, 0.71) |  | (0.25, 0.62) | (0.21, 0.47) |
| **Chronic disease** |  |  |  |  |  |
| No (ref.) |  |  |  |  |  |
| Yes | 0.31*** | 0.42*** |  | 0.64* | 0.44*** |
|  | (0.14, 0.47) | (0.26, 0.58) |  | (0.40, 1.02) | (0.30, 0.65) |
| **Social activity** |  |  |  |  |  |
| No (ref.) |  |  |  |  |  |
| Yes | -0.22*** | -018*** |  | 1.56** | 1.66*** |
|  | (-0.37, -0.07) | (-0.29, -0.07) |  | (1.09, 2.25) | (1.18, 2.34) |
| **Survey year** |  |  |  |  |  |
| 2011 (ref.) |  |  |  |  |  |
| 2015 | -0.22*** | -0.30*** |  | 1.81** | 1.66*** |
|  | (-0.40, -0.05) | (-0.40, -0.20) |  | (1.11, 2.95) | (1.17, 2.35) |
| **N** | 3882 | 3882 |  | 3882 | 3882 |
| ***Adj.R^2^/Pseudo R^2^*** | 0.23 | 0.24 |  | 0.13 |  |

CHARLS= China Health and Retirement Longitudinal Study. CHAI=Chinese Healthy Ageing Index. OLS=ordinary least squares. RE=random effects. * p≤0.1, ** p≤0.05, *** p≤0.01.
